# Quantified Head-Ball Impacts in Soccer: A Preliminary, Prospective Study

**DOI:** 10.1177/2689288X251380145

**Published:** 2025-09-25

**Authors:** Hugh McCloskey, Carolyn Beth McNabb, Pedro Luque Laguna, Bethany Keenan, John Evans, Derek K. Jones, Marco Palombo, Megan Barnes-Wood, Rhosslyn Adams, Sean Connelly, Peter Theobald

**Affiliations:** ^1^ Cardiff School of Engineering, Cardiff University, Cardiff, UK.; ^2^ Cardiff University Brain Research Imaging Centre, Cardiff University, Cardiff, UK.; ^3^ Charles Owen & Co, Wrexham, UK.; ^4^ Football Association of Wales (FIFA Medical Centre of Excellence), Pontyclun, UK.

**Keywords:** chronic traumatic encephalopathy, head impact, mild traumatic brain injury, neurodegenerative disease

## Abstract

Repetitive, sub-concussive head impacts have been associated with increased chronic traumatic encephalopathy (CTE) incidence. CTE diagnosis traditionally relies on postmortem examination, which limits precise correlation between cause and effect. This prospective study embraced innovative diffusion magnetic resonance imaging, which enables *in vivo* quantification of acute, subacute, and chronic changes in brain tissue microstructure. This approach was used to evaluate changes in white matter microstructural status at intervals up to 180 days following a specified soccer heading protocol. This study was approved by university research ethics committees. Twelve adult males were recruited to the study and gave signed, informed consent. Six Intervention participants were university-level soccer players, with six Control participants drawn from university-level noncontact sports. Multi-shell diffusion-weighted MRI data were acquired on a 3T Siemens Connectom (300 mT/m) scanner using the HARDI protocols. Baseline measures of fractional anisotropy, mean diffusivity, and mean kurtosis were acquired at day 0. The Intervention cohort then performed 10 soccer “headers” in a laboratory, with acceleration-time data captured using an instrumented mouthguard and post-processed to report common metrics. The Intervention group was then re-scanned at day 1 (*n* = 6), day 90 (*n* = 5), and day 180 (*n* = 4). The Control group was re-scanned at day 1 (*n* = 6) and day 180 (*n* = 3). Many brain tracts were identified as having significant (*p* < 0.05) changes in white matter microstructural changes at day 90, which correlated strongly with the magnitude of head impact. A smaller number of tracts had changes at day 1 and day 180. These results indicate that, within this pilot population, the magnitude of repeated soccer headers appears to correlate with the magnitude of white matter microstructural change. Additional investigation is required to determine whether the effect of such an intervention influences long-term brain health risk.Board

## Introduction

Dementia pugilistica, or “chronic traumatic encephalopathy” (CTE), is a progressive, degenerative brain disease that is typically associated with cognitive and behavioral changes.^1^ Diagnosis can only be confirmed during postmortem examination, identified by pathognomonic neurofibrillary tangles and hyperphosphorylated tau protein deposition. Presentation correlates with repeated head impacts, with more severe CTE associated with longer playing careers of elite male American football players,^2^ a sport synonymous with helmeted head collisions. CTE has also been identified in some former elite male soccer players, especially those who played in defensive positions, which traditionally demand more head-ball impacts.^3,4^ Sport governing bodies now frequently refine protocols and procedures that consider concussion and CTE risk, typically focusing on reducing head impact frequency and magnitude. As an example, the English and Welsh governing bodies for soccer (Football Association; Football Association of Wales) now advise limiting adult players to 10 “high force” headers per week, defined as a ball delivered from farther than 35 m.^5^ The validity of such a threshold in reducing CTE risk is unclear, however, given the decades-long lag between impact exposure and postmortem diagnosis.

Diffusion magnetic resonance imaging (dMRI) offers an opportunity for *in vivo* quantification of acute, subacute, and chronic changes in brain tissue microstructure.^6,7^ Innovative dMRI protocols can achieve the detection of subtle white matter microstructural changes.^8,9^ Using these data, diffusion tensor imaging (DTI) and diffusion kurtosis imaging (DKI) allow for the derivation of fractional anisotropy (FA), mean diffusivity (MD), and mean kurtosis (MK). Combining DTI and DKI enables a more detailed characterization of the diffusion-weighted signal by capturing both Gaussian and non-Gaussian diffusion components, providing greater sensitivity to microstructural alterations such as those observed in CTE. Unlike DTI, which primarily relies on lower b-value data and assumes Gaussian diffusion, the addition of DKI allows for a more comprehensive assessment of tissue microstructure.^10^ DTI studies have shown that, following mild traumatic brain injury (mTBI), FA values tend to decrease, while MD measures generally increase, suggesting a disruption to barriers hindering diffusion of water molecules.^11,12^ These changes to FA and MD may reflect axonal loss, demyelination, axonal swelling, and edema.^12–14^

DKI can detect more subtle changes in tissue compartments resulting from mTBI, by quantifying the non-Gaussian component of the diffusion displacement profile with a fourth-order kurtosis tensor and deriving rotationally invariant quantitative measures. MK is one such scalar DKI measure that quantifies the degree of non-kurtosis across all directions in a specified voxel.^15^ Axonal swelling increases the intra-axonal volume, creating a more restricted environment and leading to more non-Gaussian behavior that elevates MK.^13^ Alterations in myelin integrity and extracellular geometry introduce diffusion barriers at various scales and increased diffusion heterogeneity, while reactive astrogliosis increases the structural complexity and barriers to diffusion. Lower MK coincides with reduced FA and increased MD at the acute and subacute phases after mTBI.^16–18^

Imaging protocols provide a valuable route for quantifying white matter microstructural changes; however, surveying specific populations can have scientific, logistical, and economic barriers. Affordable, wearable sensors can now capture head kinematic data, enabling brain trauma predictions via established injury functions.^19,20^ Instrumented mouthguards (iMG) capture six degrees of freedom kinematics during sporting activity and have been widely used in sports, including soccer.^21,22^ Injury metrics including peak angular velocity (PAV), peak linear acceleration (PLA), and peak angular acceleration (PAA) are direct outputs. Post-processing with respect to the time domain produces head injury criterion (HIC) and rotational injury criterion (RIC), more accurate head trauma metrics.

This study sought to investigate the association between the *magnitude* of head injury metrics (as measured via iMG) and the *magnitude* of change in white matter microstructural status (as measured via DKI), across an age- and gender-matched population.

## Methods

### Study design and participants

A controlled cohort study was designed to longitudinally analyze the brain health of soccer players (“Intervention”) exposed to 10 headers within a controlled, laboratory environment. Six Intervention participants were recruited from a university-level soccer team. All were Caucasian, aged 21.3 ± 1 years, height = 1.78 ± 0.06 m, mass = 69.4 ± 7.6 kg. Recruitment targeted those playing positions where head impacts are less likely, to minimize in-season non-recorded exposures. Control participants (*n* = 6) were race-, age-, and gender-matched, with age = 20.7 ± 1 year; height = 1.79 ± 0.03 m, mass = 76.3 ± 7.9 kg. They were recruited from university teams, including tennis and rowing, targeted due to the low head impact prevalence. Participant exclusion criteria included sustaining a concussion during the period of study, having a prior concussion diagnosis, or any contraindication to MRI. The study was approved by Cardiff University’s Research Ethics Committees in Engineering (2020_ENGIN_PGR_PT-HM_R1) and Psychology (EC.21.03.09.6320RA). All participants provided written, informed consent.

### Patient and public involvement

The Football Association of Wales was involved in the design of this methodology. This ensured that the ball delivery distances and heading frequency represented a training scenario. It was also noted that mouthguards are not worn during soccer, and so participants could not be expected to capture data during interim training and match-play sessions. All participants understood the potential influence of any unrecorded head impacts during the study period, agreed with attempts to minimize additional head impacts, and to compile an electronic log of any exposures.

### Heading protocol and head kinematics

A laboratory-based intervention methodology was developed to replicate generalized soccer heading scenarios.^23^ Each Intervention participant headed three short (7.65 m), three medium (15.5 m), and four long (23 m) passes, spaced evenly across a 90-min test period. Individual iMGs (Protecht; Sports and Wellbeing Analytics, Swansea, UK) captured head acceleration-time traces over a period of 104 ms.^24^ The iMG comprised a 3-axis accelerometer sampling at 1 kHz (±200 g), a 3-axis gyroscope sampling at 0.952 kHz (±35 rad/sec), and an additional accelerometer with a focused bandwidth (0.5–1 kHz).^24^ Linear and rotational velocity and angular acceleration were collected in real time via Bluetooth connection, with post-processing enabling derivation of kinematic metrics including PAV, PLA, and PAA. HIC was calculated for a 15 ms time interval, while RIC was calculated over a 36 ms window.^23^

### MRI data acquisition

Multi-shell diffusion-weighted MRI data were acquired on a 3T Siemens Connectom (300 mT/m) scanner. All participants were scanned at day 0 (Intervention = October 2021, Control = April 2022). The six Intervention participants then performed 10 headers before being re-scanned on day 1 (mean time after intervention = 22 ± 3 h), day 90 ± 7, and day 180 ± 7. The Control group was re-scanned on day 1 and day 180 ± 7.

High angular resolution data were acquired over multiple shells according to a previously reported protocol.^8^ This took 18 min, using a single-shot spin-echo, echo-planar imaging sequence, with the following directions: b = 200 sec/mm^2^ (20 directions); b = 500 sec/mm^2^ (20 directions); b = 1200 sec/mm^2^ (30 directions), and three shells of 61 directions each at b = 2400, 4000, and 6000 sec/mm^2^. A reverse phase encode, posterior to anterior (P ≫ A) dataset was also acquired for distortion correction, comprising two non-diffusion-weighted images and b = 1200 sec/mm^2^ (30 directions). Data acquisition details for all b values are as follows: time relaxation (TR) = 3000 ms; time excitation (TE) = 59 ms; field of view (FOV) 220 × 200 × 132 mm^3^; matrix size 110 × 110 × 66; voxel size 2 × 2 × 2 mm^3^; in-plane acceleration (GRAPPA) factor of 2. Diffusion gradient duration and separation were 7 and 24 ms, respectively.

### Data processing and quality assessment

#### Preprocessing

Preprocessing rectifies distortion and artifacts within the MRI data. All non-brain data were excluded by creating a mask of the first non-diffusion-weighted image from each phase-encoding direction, anterior to posterior (A ≫ P) and P ≫ A, using the FMRIB Software Library’s Brain Extraction Tool (FSL, version 6.0.3).^25^ Noise level assessment and noise reduction in the diffusion MRI data were performed using MRtrix3 (www.mrtrix.org), based on principal component analysis (MP-PCA).^26–28^ Drifts in scanner performance (and therefore image intensity) were corrected by adjusting the diffusion-weighted MRI data to align with temporally distributed b0 sec/mm^2^ (non-diffusion-weighted) images, which were dispersed over time, using a tailor-made MATLAB code (MATLAB R2017b; MathWorks Inc., Natick, MA). The Slicewise OutLIer Detection (SOLID) approach was also applied, adopting 3.5 and 10 thresholds based on a modified Z-score and a variance-focused intensity metric.^29^ Adjustment of susceptibility-induced off-resonance fields was deduced from the b0 data acquired in both A ≫ P and P ≫ A phase-encoding directions. The necessary corrections for these fields, as well as for the distortions caused by Eddy currents and movements of the subject, were effected through Topup^30,31^ and Eddy^32^ functionalities (FSL, version 6.0.3). Distortions from gradient nonlinearity were addressed through another custom-developed MATLAB code. Attenuation of Gibbs ringing artifacts was corrected using a local sub-voxel-shift technique,^28,33^ before computing fiber orientation distribution function (fODF; both in MRtrix3) to leverage a multi-shell, multi-tissue constrained spherical deconvolution (CSD) method.^28,34^

#### Quality assurance

A visual inspection was conducted to identify and discard any data with observable artifacts or improper reconstruction. Head motion was estimated between each pair of consecutively acquired volumes and relative to the first volume in the scan, considering translational and rotational motion in three-dimensional space.^35^ Data were excluded if displacement exceeded 5.0 mm when compared with the first volume after motion correction. Additionally, the average values of translational and rotational motion were calculated for each subject, with exclusion for those with average motion metrics exceeding three standard deviations.

### Post-processing

The diffusion kurtosis representation was fitted to the shells up to b = 2400 sec/mm^2^ using the Dipy Dkifit model, which fits the diffusion kurtosis model and then computes the diffusion tensor metrics.^10,36^ This process outputs FA, MD, and MK maps for each subject at each timepoint. The preprocessed diffusion data from each participant’s first timepoint were used to generate bundle segmentations, ending segmentations, and tract orientation maps. TractSeg, a convolutional neural network-based method, was employed for this purpose.^37^ Briefly, fields of fODF peaks were extracted from the preprocessed diffusion MRI data and estimated using the multi-shell, multi-tissue CSD method, implemented in MRtrix3.^34^ Orientation maps describing fiber orientation within 50 segmented tracts were derived from the same fODF data and incorporated into the segmentation outputs. These are detailed in Supplementary Table S1. Bundle-specific tractograms were then generated using the segmented bundles from the initial timepoint and the fiber direction information derived from the orientation maps. Quantitative tractometry analysis was then performed on the white matter tractograms and parametric maps in TractSeg, using a methodology developed in Chandio et al.^38^ The tractometry analysis reports metric values at 98 segments along each length.

### Statistical analysis

Linear mixed-effects model (LMEM) analyses were performed using R (version 4.0.3, www.r-project.org), LmerTest, and lmerPerm 0.1.9.^39^ Pearson’s and Spearman’s correlation, and linear regression, were performed using Python (version 3.8.13, www.python.org) with the scipy.stats package.^40^

#### Linear mixed-effects modeling

An LMEM was fitted to the data using the LmerTest package, enabling direct comparison with variation at different timepoints across the Control and Intervention groups, including the interaction between timepoints and group memberships, while accounting for random intercepts per subject. Ten thousand permutation tests were then performed to estimate *p* values for the fixed effects (lmerPerm 0.1.9).^39^ *p* Values for each tract were corrected for multiple comparisons based on the number of tracts using the Bonferroni method, due to the small sample size and risk of type II error.

#### Longitudinally correlating the magnitude of head impact with microstructural change

The mean and median FA, MD, and MK across all tracts were calculated, then the difference between two timepoints was computed to provide a “mean delta” and a “median delta,” in addition to the peak and mean kinematic metrics, for each participant. Potential relationships were analyzed through visualization using scatter plots (generated by the matplotlib library^41^), linear regression, parametric Pearson’s correlation, and nonparametric Spearman’s rank correlation (employing the scipy.stats.linregress, scipy.stats.pearsonr, and scipy.stats.spearmanr functions^42^). Pearson’s correlation coefficient (*r*), Spearman’s rho, coefficient of determination (*R*^2^), and associated *p* values were calculated for each linear fit.

## Results

The Control and Intervention groups were both MRI scanned at day 0 and day 1. The Intervention group underwent additional MRI scans at day 90 and day 180. One day 90 dataset was excluded due to the excess head movement, and two participants did not attend the day 180 scan. Three participants from the Control group attended the day 180 scan. This is all described pictorially in Supplementary Figure S1. None of the participants reported any other head impact activity across the investigated period.

### Fractional anisotropy

Raw FA results from the LMEM analysis are presented in Supplementary Figure S2.

**FIG. 1.**
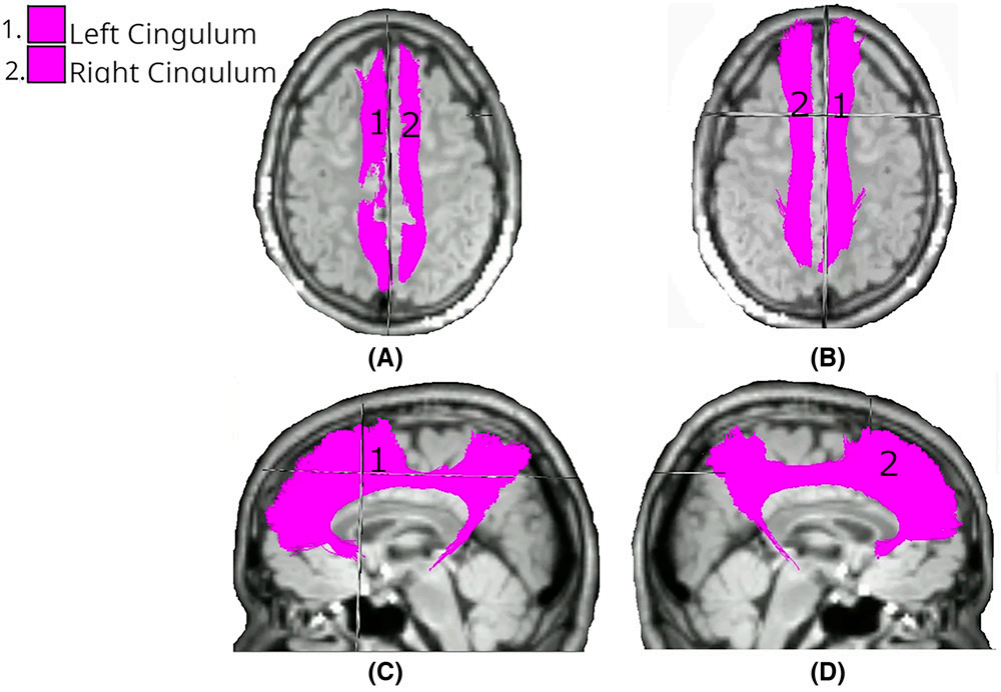
White matter tracts showing significant differences in FA changes between Intervention and Control groups at day 1. Colored regions overlaid on T1-weighted MRIs indicate tracts with statistically significant differences (*p* < 0.05), based on LMEM. **(A)** Superior axial view; **(B)** inferior axial view; **(C)** left midsagittal view; **(D)** right midsagittal view. FA, fractional anisotropy; LMEM, linear mixed-effects model.

Figure 1 describes the significant results of the LMEM, comparing the mean FA changes in the Intervention group at day 1 across all tracts. Significant differences (*p* < 0.05) were identified in the right and left cingulum (CG).

**FIG. 2.**
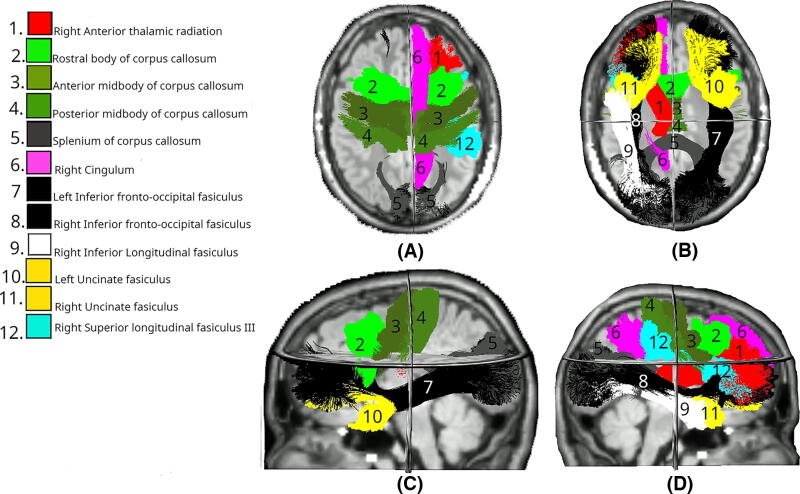
White matter tracts showing significant differences in FA changes between Intervention (day 0–90) and Control (day 0–180) groups. Colored regions overlaid on T1-weighted MRIs indicate tracts with statistically significant differences (*p* < 0.05), based on LMEM. **(A)** Superior axial view; **(B)** inferior axial view; **(C)** left midsagittal view; **(D)** right midsagittal view. FA, fractional anisotropy; LMEM, linear mixed-effects model.

The FA was significantly lower in multiple white matter tracts at day 90 between the Intervention and Control groups (Fig. 2). Bilateral reductions were observed in the uncinate fasciculus (UF; left: *p* = 0.010, right: *p* < 0.01) and inferior fronto-occipital fasciculus (IFO; both left and right: *p* < 0.01). Considering the commissural tracts that cross hemispheres, significant differences were found in multiple segments of the corpus callosum (CC), specifically CC_3 (*p* = 0.036), CC_4 (*p* < 0.01), CC_5 (*p* < 0.01), and CC_7 (*p* < 0.01). The right hemisphere showed changes in the anterior thalamic radiation (ATR; *p* < 0.01), inferior longitudinal fasciculus (ILF; *p* < 0.01), and superior longitudinal fasciculus III (SLF_III; *p* = 0.029). The spatial distribution of these changes suggests a particularly strong involvement of association fibers, especially those connecting frontal and temporal regions, along with substantial involvement of interhemispheric connections through the CC and cerebellar pathways.

All changes identified in the Intervention group had resolved by day 180, with no significant differences between groups in any of the tracts.

### Mean diffusivity

Raw MD results from the LMEM analysis are presented in Supplementary Figure S3.

**FIG. 3.**
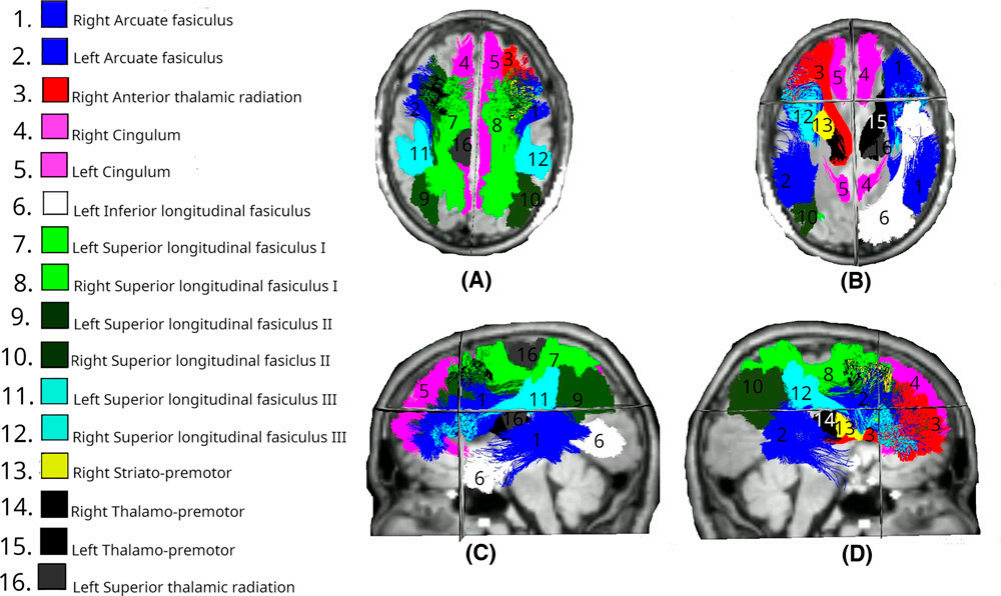
White matter tracts showing significant differences in MD changes between Intervention and Control groups at day 1. Colored regions overlaid on T1-weighted MRIs indicate tracts with statistically significant differences (*p* < 0.05), based on LMEM. **(A)** Superior axial view; **(B)** inferior axial view; **(C)** left midsagittal view; **(D)** right midsagittal view. MD, mean diffusivity; LMEM, linear mixed-effects model.

Day 1 MD data revealed significant differences in more than one-third of all tracts (Fig. 3). Bilateral differences were identified in the SLF across all three segments (SLF_I, SLF_II, and SLF_III; all *p* < 0.01), CG (both left and right: *p* < 0.01), and arcuate fasciculus (AF; left: *p* < 0.01, right: *p* = 0.018). Temporal projections showed bilateral involvement with significant differences in the temporal premotor tract (T_PREM; left: *p* = 0.032, right: *p* = 0.013). Among unilateral tracts, changes were observed in the right ATR (*p* < 0.01). The left hemisphere showed changes in the ILF (*p* < 0.01), superior temporal premotor tract (ST_PREM; *p* < 0.01), and superior thalamic radiation (STR; *p* = 0.029). The spatial distribution of these changes again suggests widespread involvement of association fibers.

**FIG. 4.**
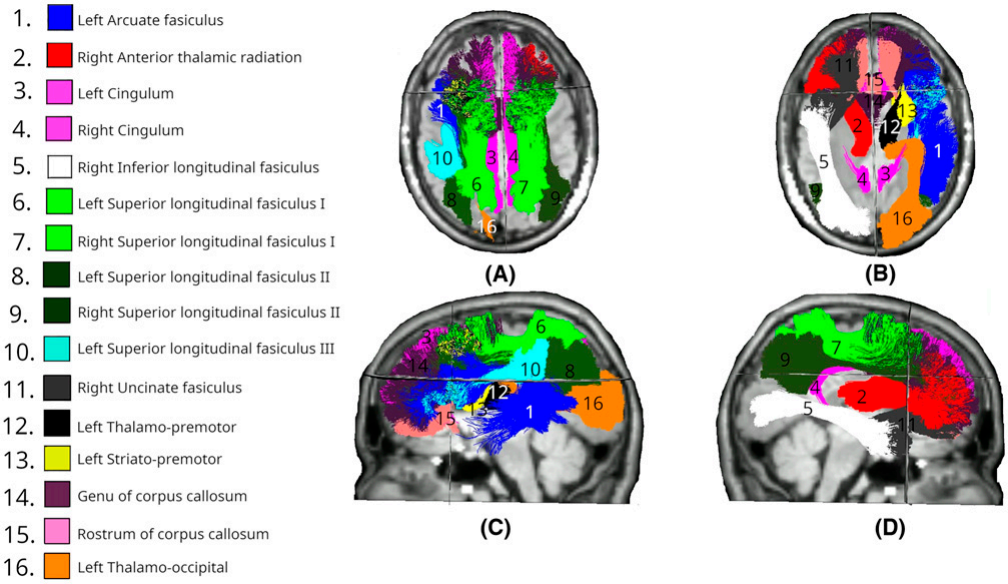
White matter tracts showing significant differences in MD changes between Intervention (day 0–90) and Control (day 0–180) groups. Colored regions overlaid on T1-weighted MRIs indicate tracts with statistically significant differences (*p* < 0.05), based on LMEM. **(A)** Superior axial view; **(B)** inferior axial view; **(C)** left midsagittal view; **(D)** right midsagittal view. MD, mean diffusivity; LMEM, linear mixed-effects model.

Day 90 data revealed significant differences across 17 tracts, maintaining the bilateral involvement of the SLF_I and CG systems and anterior commissural fibers observed on day 1, though becoming more asymmetrical at day 90 (Fig. 4). Bilateral changes were observed in the SLF_I (both *p* < 0.01), CG (both *p* < 0.01), and anterior temporal projections, with both T_PREM and ST_PREM being affected (left: both *p* < 0.01). Significant differences were found in anterior segments of the CC (CC_1: *p* = 0.011, CC_2: *p* = 0.008). The right hemisphere showed changes in the ATR (*p* < 0.01), ILF (*p* < 0.01), UF (*p* < 0.01), and SLF_II (*p* = 0.033). The left hemisphere showed changes in the AF (*p* < 0.01), SLF_II (*p* < 0.01), SLF_III (*p* < 0.01), and temporal occipital tract (T_OCC; *p* = 0.027).

**FIG. 5.**
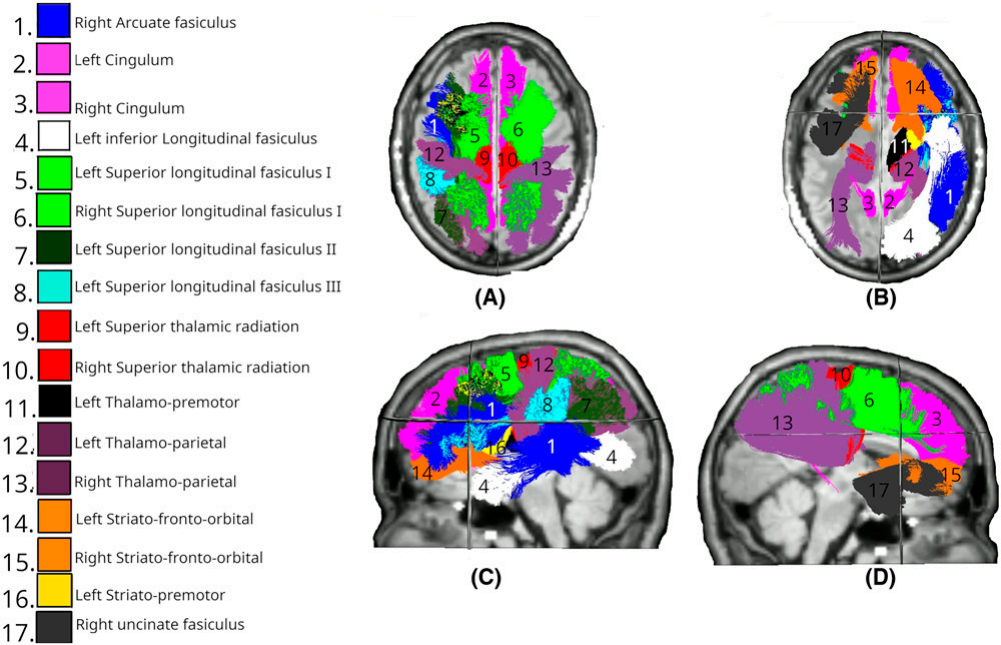
White matter tracts showing significant differences in MD changes between Intervention and Control groups at day 180. Colored regions overlaid on T1-weighted MRIs indicate tracts with statistically significant differences (*p* < 0.05), based on LMEM. **(A)** Superior axial view; **(B)** inferior axial view; **(C)** left midsagittal view; **(D)** right midsagittal view. MD, mean diffusivity; LMEM, linear mixed-effects model.

Eighteen tracts showed a significant MD difference at day 180, the greatest number of the measured period (Fig. 5). These showed both persistence and evolution of patterns seen at earlier timepoints. Bilateral changes remained in the SLF_I (both *p* < 0.01), CG (both *p* < 0.01), superior temporal fronto-occipital tract (ST_FO; both *p* < 0.01), and temporal parietal tract (T_PAR; both *p* < 0.01). The right hemisphere showed changes in the UF (*p* = 0.026) and STR (*p* = 0.037). The left hemisphere showed extensive changes in the AF (*p* < 0.01), ILF (*p* < 0.01), SLF_II (*p* < 0.01), SLF_III (*p* < 0.01), T_PREM (*p* < 0.01), STR (*p* < 0.01), and ST_PREM (*p* = 0.014). Compared with earlier timepoints, this pattern maintains the bilateral involvement of SLF_I and CG systems seen at both day 1 and day 90, though it shows a marked shift toward left hemisphere dominance in the SLF system. The anterior commissural changes seen at day 90 are no longer present, suggesting a dynamic reorganization of white matter over time.

### Mean kurtosis

Raw MK results from the LMEM analysis are presented in Supplementary Figure S4.

Analysis of day 1 data revealed a highly focused pattern of significant differences, with changes observed only bilaterally in the CG (both left and right: *p* < 0.01). This is the same pattern as day 1 FA (Fig. 1).

**FIG. 6.**
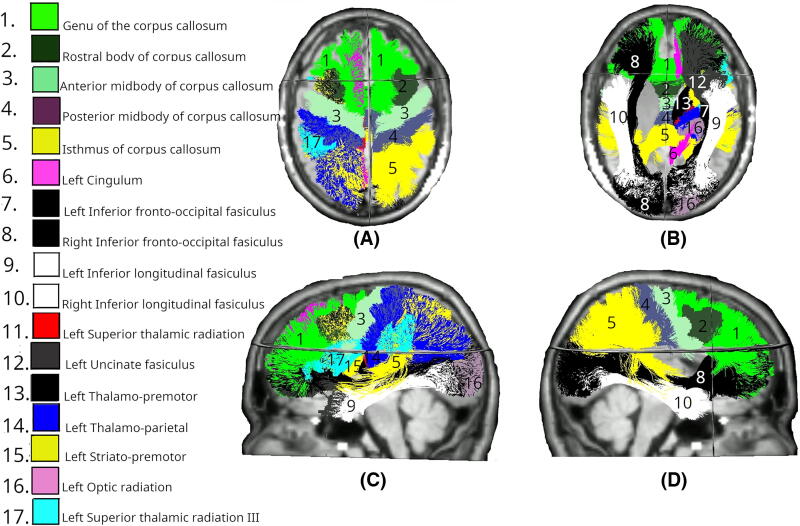
White matter tracts showing significant differences in MK changes between Intervention (day 0–90) and Control (day 0–180) groups. Colored regions overlaid on T1-weighted MRIs indicate tracts with statistically significant differences (*p* < 0.05), based on LMEM. **(A)** Superior axial view; **(B)** inferior axial view; **(C)** left midsagittal view; **(D)** right midsagittal view. MK, mean kurtosis; LMEM, linear mixed-effects model.

By day 90 (Fig. 6), 17 tracts had significant MK differences. Bilateral changes were observed in the IFO (both *p* < 0.01) and ILF (both *p* < 0.01). Extensive involvement of the CC was also noted, including CC_2 (*p* < 0.01), CC_3 (*p* < 0.01), CC_4 (*p* < 0.01), CC_5 (*p* < 0.01), and CC_6 (*p* < 0.01). For unilateral tracts, no right hemisphere tracts had a significant difference. Conversely, many left hemisphere tracts showed changes, including the UF (*p* < 0.01), STR (*p* < 0.01), T_PREM (*p* < 0.01), T_PAR (*p* = 0.006), ST_PREM (*p* = 0.008), optic radiation (OR; *p* = 0.034), SLF_III (*p* = 0.034), and CG_left (*p* < 0.01). This pattern shows interesting convergence with both FA and MD findings at day 90, particularly in the involvement of the IFO and commissural fibers, though MK demonstrates unique sensitivity to changes in visual and temporal projection pathways. The expansion from the focused day 1 pattern suggests that while early changes in tissue complexity are limited to the limbic system, by day 90 there is extensive reorganization of tissue microstructure across multiple networks, particularly in the left hemisphere.

**FIG. 7.**
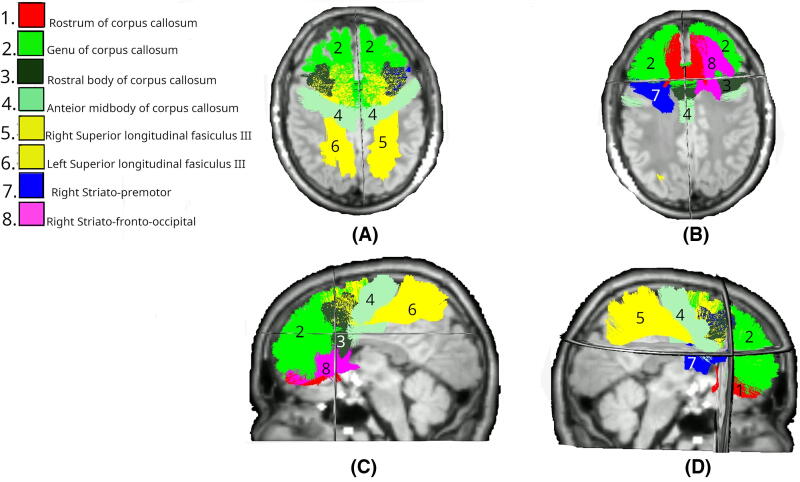
White matter tracts showing significant differences in MD changes between Intervention and Control groups at day 180. Colored regions overlaid on T1-weighted MRIs indicate tracts with statistically significant differences (*p* < 0.05), based on LMEM. **(A)** Superior axial view; **(B)** inferior axial view; **(C)** left midsagittal view; **(D)** right midsagittal view. MD, mean diffusivity; LMEM, linear mixed-effects model.

Day 180 MK data identified eight significant tracts, though with a different spatial pattern from day 1 and day 90 (Fig. 7). Among commissural tracts that cross hemispheres, involvement of the CC remained prominent but shifted anteriorly, with significant differences in CC_1 (*p* = 0.035), CC_2 (*p* < 0.01), CC_3 (*p* = 0.014), and CC_4 (*p* = 0.026). Bilateral changes were limited to the SLF_I (left: *p* = 0.033, right: *p* < 0.01). Unilateral changes were again absent from the right hemisphere, though observed in the left superior temporal fronto-occipital tract (*p* = 0.015). This pattern shows marked deviation from the extensive bilateral changes seen at day 90 in the IFO, ILF, and temporal projection pathways, suggesting a dynamic reorganization of tissue microstructure over time. Compared with MD at the same timepoint—which showed extensive left-lateralized changes in the SLF system and bilateral involvement of temporal projections—the MK changes are more focused on anterior commissural fibers. The evolution from bilateral CG involvement at day 1, through widespread changes at day 90, to this more focused pattern at day 180 suggests a complex temporal progression of microstructural reorganization, with different white matter systems showing distinct trajectories of change over time.

### Kinematic analysis

Post-processed data describe the peak values, and the cumulative peak values, for each participant (see Supplementary Fig. S5). In most instances, the peak and cumulative metrics share common trends: participant 3 scored highest across all metrics and so was expected to have the highest trauma risk; participant 4 generally achieved lower metrics and so, assuming all anatomical and physiological variables were broadly comparable across participants, would have a lower injury risk. Participant 2 is notable for their relatively high linear metrics, while scoring relatively low rotational metrics.

### Correlating kinematic injury metrics and DKI metrics

Combining these datasets allows investigation of the effect of head impact magnitude on the longitudinal changes in white matter microstructural status via DKI metrics. DKI metrics are correlated with different kinematic injury metrics at day 1, 90, and 180.

**FIG. 8.**
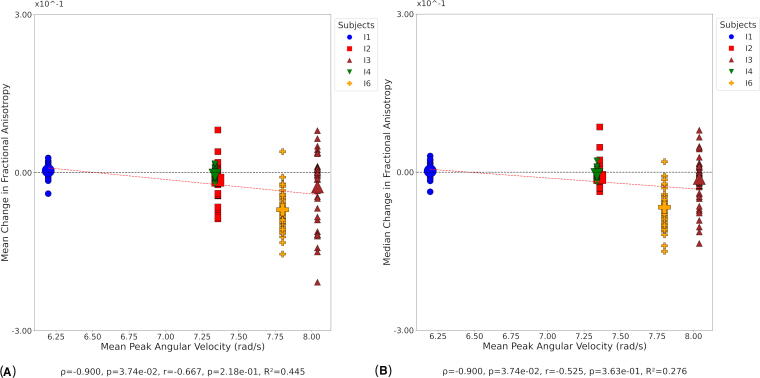
Correlation between FA and kinematic metrics at day 90. Small symbols indicate FA change in individual white matter tracts at the participant level; large symbols indicate overall change in FA for each participant. Statistical significance was assessed via Pearson’s correlation (*r*), Spearman’s correlation (*ρ*), and coefficient of determination (*R*^2^). **(A)** Mean change in FA versus mean PAV. **(B)** Median change in FA versus mean PAV. FA, fractional anisotropy; PAV, peak angular velocity.

No significant correlations were found between mean and median change in FA and any kinematic metric at day 1. At day 90, a significant negative correlation existed between mean (Fig. 8A) and median (Fig. 8B) change in FA, and mean PAV. The significant negative trends indicate that higher mean PAV values during the intervention corresponded with greater reductions in both mean and median FA. Persistent correlations between FA and kinematic metrics existed at day 180, including a strong negative relationship between mean change in FA and mean HIC15 (Supplementary Fig. S6A) and between median change in FA and mean HIC15 (Supplementary Fig. S6B). Additional significant negative correlations were observed between mean and median FA changes and mean PAV at day 180 (Supplementary Fig. S6C,D).

**FIG. 9.**
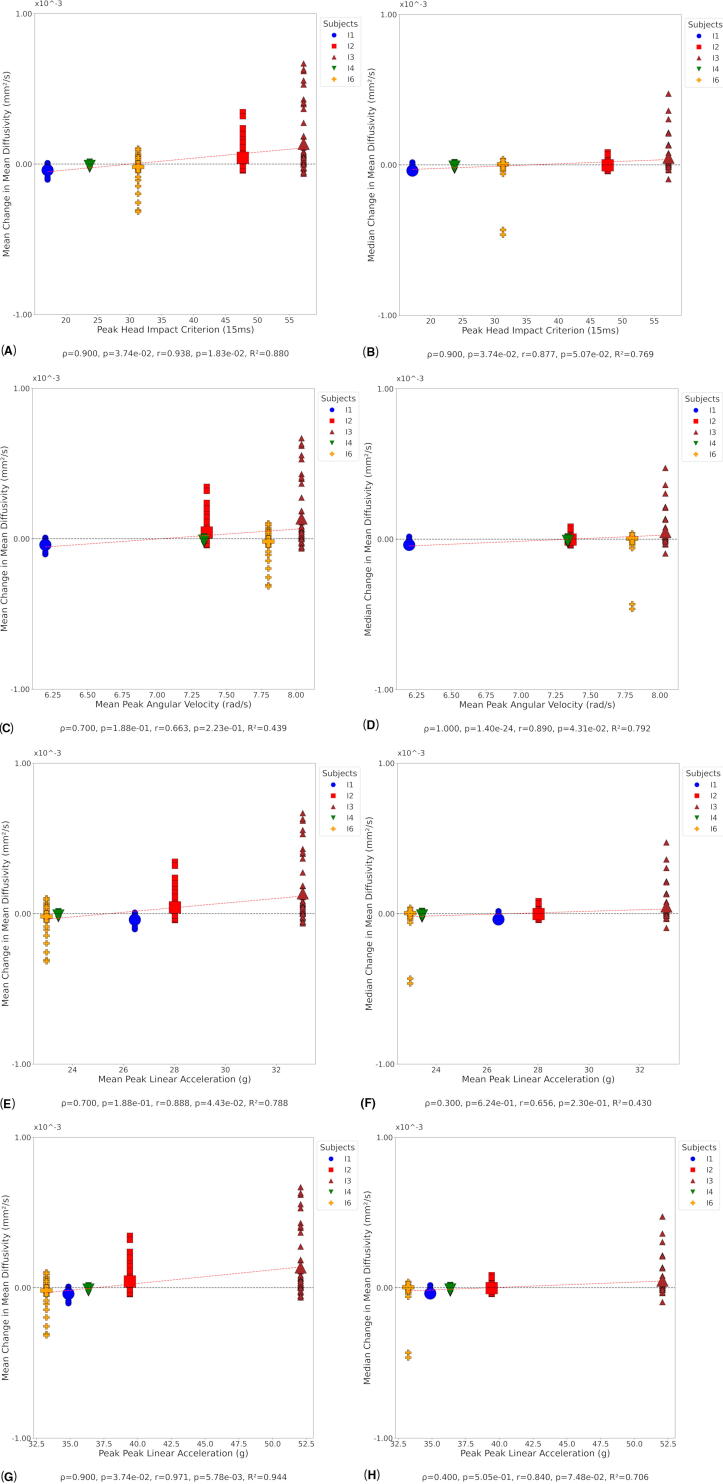
Correlation between MD and kinematic metrics at day 90. Small symbols indicate MD change in individual white matter tracts at the participant level; large symbols indicate overall change in MD for each participant. Statistical significance was assessed via Pearson’s correlation (*r*), Spearman’s correlation (*ρ*), and coefficient of determination (*R*^2^). **(A)** Mean change in MD versus peak HIC. **(B)** Median change in MD versus peak HIC. **(C)** Mean change in MD versus mean PAV. **(D)** Median change in MD versus mean PAV. **(E)** Mean change in MD versus mean PLA. **(F)** Median change in MD versus mean PLA. **(G)** Mean change in MD versus peak PLA. **(H)** Median change in MD versus peak PLA. MD, mean diffusivity; HIC, head injury criterion; PAV, peak angular velocity; PLA, peak linear acceleration.

No significant correlation was evident at day 1 between mean change in MD and peak PLA (Supplementary Fig. S7A), though there was a significant negative correlation between median change in MD and peak PLA (Supplementary Fig. S7B). At day 90, several significant positive correlations were observed between mean (Fig. 9A) and median (Fig. 9B) change in MD and peak HIC, mean (Fig. 9C) and median (Fig. 9D) change in MD and mean PAV. Additional significant correlations were found between mean (Fig. 9E) and median (Fig. 9F) change in MD and mean PLA, and mean (Fig. 9G) and median (Fig. 9H) change in MD and peak PLA. At day 180, significant positive correlations were observed between mean change in MD and mean HIC (Supplementary Fig. S8A), and median change in MD and mean HIC (Supplementary Fig. S8B). Strong positive correlations were also found between mean change in MD and peak HIC (Supplementary Fig. S8C), and median change in MD and peak HIC (Supplementary Fig. S8D). Additionally, positive Spearman correlations were observed between mean change in MD and mean PAV (Supplementary Fig. S8E) and median change in MD and mean PAV (Supplementary Fig. S8F).

**FIG. 10.**
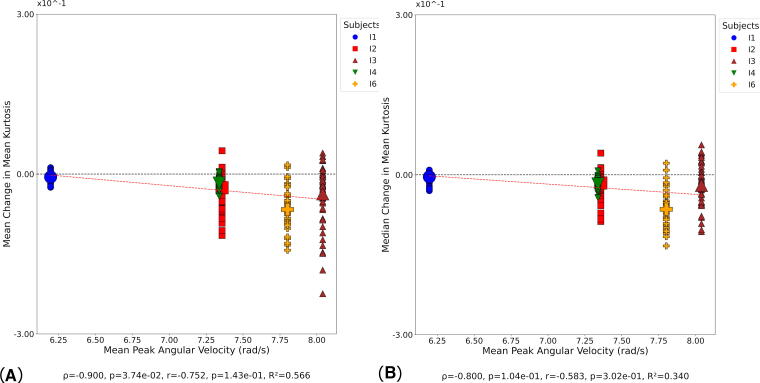
Correlation between MK and kinematic metrics at day 90. Small symbols indicate MK change in individual white matter tracts at the participant level; large symbols indicate overall change in MK for each participant. Statistical significance was assessed via Pearson’s correlation (*r*), Spearman’s correlation (*ρ*), and coefficient of determination (*R*^2^). **(A)** Mean change in MK versus mean PAV. **(B)** Median change in MK versus mean PAV. MK, mean kurtosis; PAV, peak angular velocity.

At day 1, significant positive correlations were observed between mean change in MK and mean PAA (Supplementary Fig. S9A), while no significant correlation was found for median change in MK and mean PAA (Supplementary Fig. S9B). A significant correlation was observed between mean change in MK and peak PAA (Supplementary Fig. S9C), while median change in MK showed no significant correlation (Supplementary Fig. S9D). At day 90, a significant negative correlation was observed between mean change in MK and mean PAV (Fig. 10A), while no significant correlation was found between median change in MK and mean PAV (Fig. 10B). Both correlations showed moderate to strong negative relationships based on Spearman’s rho, though only the mean change reached statistical significance, while neither Pearson correlation achieved significance. No significant correlations were observed between MK and any kinematic metric at day 180.

## Discussion

This study identified significant differences in changes to DKI metrics in the Intervention versus Control groups, tested via LMEM following an intervention of 10 headers, measured across a common period. This study also found statistically significant correlations between the magnitude of kinematic injury metrics and the change in diffusion measures. This was most pronounced at day 90.

LMEM analysis of FA and MK at day 1 demonstrated no statistical change versus the Control group in all tracts except the bilateral CG. This lack of change at the acute stage is consistent with previous findings.^43^ LMEM analysis of MD at day 1 showed significant decreases in the bilateral SLF. An MD decrease in the SLF has previously been observed in concussed athletes.^44^ Significant decreases were also seen in the ATR and STR tracts, a pattern consistent with athletes exposed to collisions during sport.^45^ The decreasing MD at day 1 coincides with concomitant MD increases in the Control group, which may confound drawing inferences from this change.

The subacute (day 90) timepoint LMEM analysis showed statistical decreases in MK and FA, and statistical increases in MD across many tracts. The spatial location of the changes at this timepoint, with the CC and SLF shown to be preferentially affected, is consistent with patterns of changes associated with mTBI and TBI.^45–48^

MK data showing a more marked decrease at day 90, following a slight increase at day 1, is consistent with changes observed in concussed athletes.^49^ Such changes have been proposed to represent reactive astrogliosis in animal models.^50^ This MK decrease is also reflective of the trend seen in concussed athletes in the subacute phase after injury.^18,51^

Intervention FA returned to baseline at day 180 (chronic phase), with no significant differences versus the Control data found in LMEM analysis.

Lower MD and higher MK were identified in many tracts at day 180, coinciding with those that changed at day 90 (subacute phase), including the SLF and ILF in MD, and CC and SLF in MK. This biphasic response in MD and MK between the subacute and chronic phase in the CC is consistent with a trend observed in other mTBI cases.^17^

Tracts of the CC, particularly the most anterior regions, appeared most significantly influenced by the intervention across the three DKI metrics and across all timepoints, which is consistent with studies reporting concussive and sub-concussive head impacts.^17,52^ These patterns in MD and MK have previously been cited as the two most important metrics for detecting repetitive sub-concussive head injuries in athletes,^49^ and the tracts most likely to be affected were the CC and caudal tracts such as the SLF and ILF.

In the current study, HIC15 emerged as the kinematic metric that produced the strongest, and the most, correlations, specifically between peak HIC and MD change at day 90 (*R*^2^ = 0.880) and day 180 (*R*^2^ = 0.990). This is consistent with findings from the automotive sector.^53^ Mean PAV produced the greatest number of significant results across all metrics and timepoints. This is consistent with PAV reported elsewhere.^54^ The kinematic metrics presented in this study are likely a conservative representation of soccer exposures, due to all passes <30 m, a threshold defining “high energy passes.”

Drawing comparisons between the kinematic values presented in this study and data reported in other studies is challenging, due to variations in measurement protocols and different levels of player training and skill.^23,55^ Some studies used iMGs to capture head kinematics of university-level males (aged 21.3 ± 1.89) and females (aged 21.25 ± 1.68) with previous soccer experience. The protocol (distance = ∼12.2 m, ball velocity = 11.18 ms^−1^, ball pressure = 0.59 bar) resulted in 78% lower PLA values and 43% lower PAA values compared with those reported at a similar velocity in this study. Although the protocol setup was similar, the lower values observed are likely due to the reduced ball inflation pressure.^56^ Another study used skin patch sensors to capture on-field PLA and PAA values in semi-professional female soccer players. PLA for distances represented in this study (5–20 m) were similar (26.9 g); however, PAA values were much higher (5659.8 rad/sec^2^). The use of skin-mounted accelerometers may have contributed to their notably elevated PAA values, as studies indicate frequent occurrence of artifactual data caused by soft tissue deformation. Additionally, it has been observed that females exhibit greater linear and angular acceleration compared with their male counterparts, likely attributable to a lower head mass. Head injury risk metrics varied significantly in youth male footballers (aged 14.8 years), based on ball delivery distance.^57^ Headers delivered from 15 m, simulating in-game scenarios such as a corner kick, resulted in the highest PLA and PAV. Specifically, the attacking headers produced the greatest head impact magnitudes, likely due to the additional power and direction applied to the ball. In contrast, headers from 5 m, performed either with the feet grounded or while jumping, produced significantly lower PLA and PAV.^58^

### Limitations

The data presented in this study were captured from a very small cohort (*n* = 12), with analysis and interpretation limited further by participant attrition across the 180-day follow-up. The results are also from a homogeneous population of Caucasian adult males, which, while intentionally constraining variables, does prevent translation of these findings to consider younger and/or female players. Participant, facility, operator, and financial constraints meant that follow-up dMRI scans were infrequent, though they still represent similar intervals to other studies. That Control timepoints were fewer than the Intervention group is consistent too; hence, this study presents a valid and meaningful approach to garner an appreciation of the correlation between head impact magnitude and changes to white matter microstructural status.

## Conclusions

Statistically significant correlations have been identified between the magnitude of head impacts and the magnitude of change in white matter microstructural status from day 1 to day 180. Kinematic data were interpreted to represent a relatively modest head impact, though these findings indicate that even these can cause brain changes extending for many months. Aside from sampling larger and more diverse populations, a further step is to determine how cumulative head impacts map onto these correlations, which could provide a greater understanding of the neuropathology underpinning CTE.

## Transparency, Rigor, and Reproducibility

Twelve participants were recruited for this study. All participants were MRI scanned on day 0 and day 1. The 6 Intervention participants were exposed to 10 headers on day 0. Intervention participants were then invited for MRI scanning at day 90, though participant I5 did not attend. Intervention participants were also invited to attend MRI scanning at day 180, though participants I3 and I4 did not attend. The Control participants were invited to attend MRI scanning at day 180, though 3 (C7, C9, C12) did not attend. Participant head kinematics were recorded during the intervention using personalized, instrumented mouthguards. All denture impressions were performed by the same dental surgeon, and all mouthguards were manufactured by the same company. All kinematic data were processed using standard methods to compute established head injury metrics. All MRI data were processed using a linear mixed-effects model. Data from this study are available in the Cardiff University repository, with a hyperlink to follow and under a Creative Commons Attribution license (CC-BY 4.0).

## Abbreviations Used

A>>P: Anterior-to-posterior (phase-encode direction)
AF: Arcuate fasciculus
ATR: Anterior thalamic radiation
b0: Non-diffusion-weighted (b = 0) images
CC_1: Corpus callosum rostrum
CC_2: Corpus callosum genu
CC_3: Corpus callosum rostral body (premotor)
CC_4: Corpus callosum anterior midbody (primary motor)
CC_5: Corpus callosum posterior midbody (primary somatosensory)
CC_6: Corpus callosum isthmus
CC_7: Corpus callosum splenium
CG: Cingulum
CSD: Constrained spherical deconvolution
CTE: Chronic traumatic encephalopathy
dMRI: Diffusion magnetic resonance imaging
DKI: Diffusion kurtosis imaging
DTI: Diffusion tensor imaging
FA: Fractional anisotropy
FOV: Field of view
FSL: FMRIB Software Library
fODF: Fibre orientation distribution function
GRAPPA: Generalized autocalibrating partially parallel acquisitions
HARDI: High angular resolution diffusion imaging
HIC: Head injury criterion
HIC15: HIC computed over a 15 ms window
iMG: Instrumented mouthguard
IFO: Inferior fronto-occipital fasciculus
ILF: Inferior longitudinal fasciculus
KESS: Knowledge Economy Skills Scholarship
LMEM: Linear mixed-effects model
MD: Mean diffusivity
MK: Mean kurtosis
MP-PCA: (Marchenko–Pastur) principal component analysis †
MRI: Magnetic resonance imaging
OR: Optic radiation
P>>A: Posterior-to-anterior (phase-encode direction)
PAA: Peak angular acceleration
PAV: Peak angular velocity
PLA: Peak linear acceleration
R²: Coefficient of determination
RIC: Rotational injury criterion
SLF: Superior longitudinal fasciculus
SLF_I: Superior longitudinal fasciculus I
SLF_II: Superior longitudinal fasciculus II
SLF_III: Superior longitudinal fasciculus III
SOLID: Slicewise OutLIer Detection
ST_FO: Superior temporal frontal-occipital tract
ST_PREM: Superior temporal premotor tract
STR: Stratum/Superior thalamic radiation ‡
T_OCC: Temporal occipital tract
T_PAR: Temporal parietal tract
T_PREM: Temporal premotor tract
TBI: Traumatic brain injury
TE: Echo time
TR: Repetition time
UF: Uncinate fasciculus
UKRI: UK Research and Innovation
