# Supplementary material for: Quantified Head-Ball Impacts in Soccer: A Preliminary, Prospective Study
Source: Neurotrauma Rep. 2025 Sep 25;6(1):928–43. doi: 10.1177/2689288X251380145 (PMC12547406; doi:10.1177/2689288X251380145)
Supplement: Supplementary Figures [file 2689288x251380145_supplementary_figures.pdf]

SUPPLEMENTARY MATERIAL

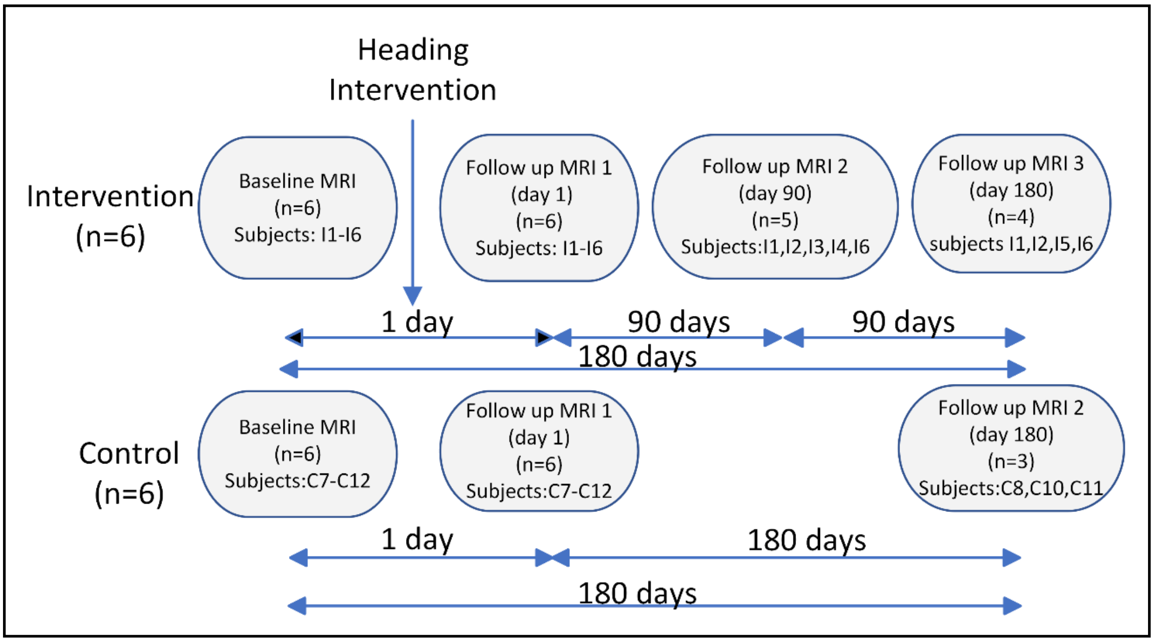

Supplementary Figure 1

Pictorial explanation of participant engagement and retention, relative to scanning sessions.

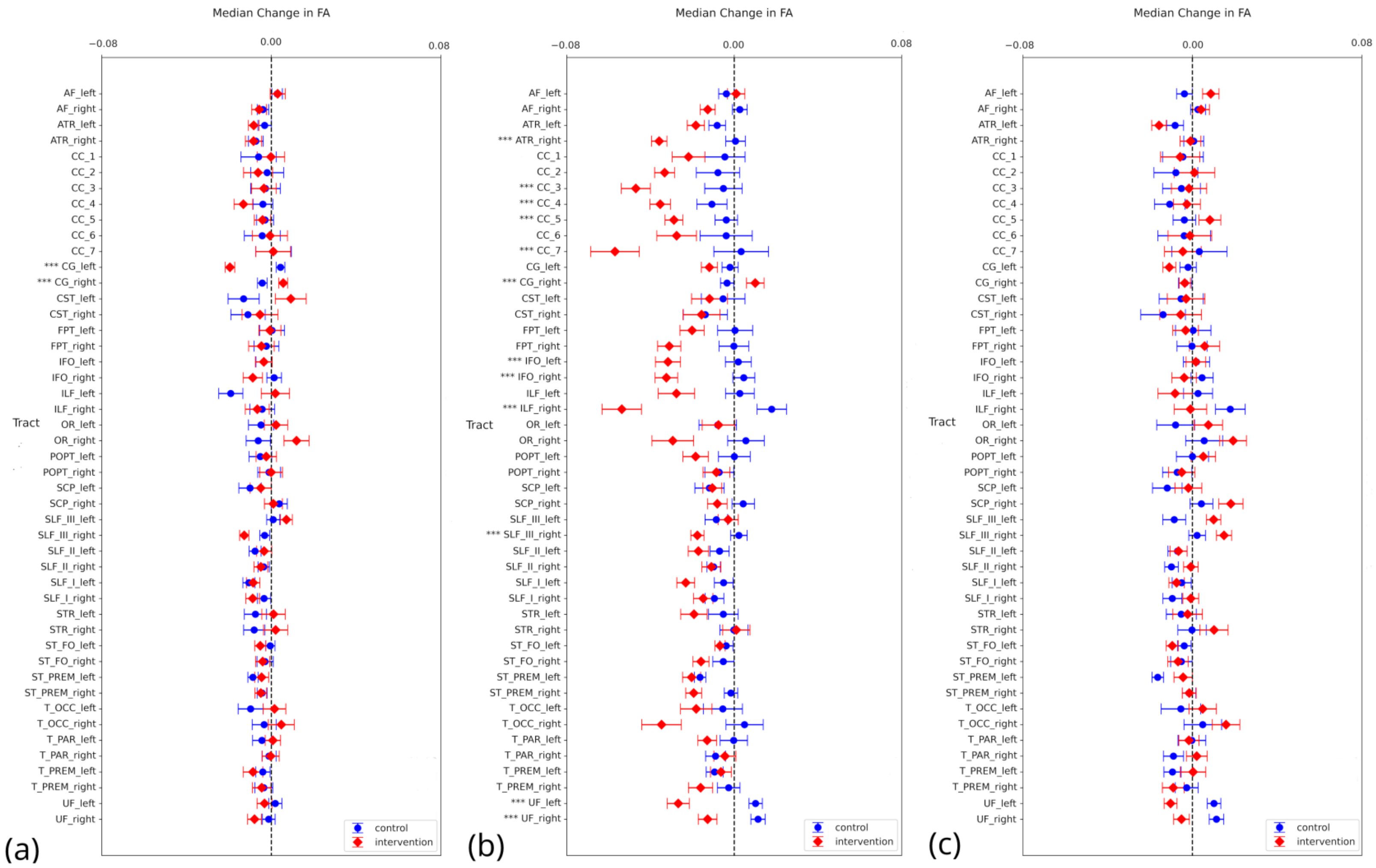

Supplementary Figure 2

Variation in FA between day 1 (a), day 90 (b) and day (180), relative to day 0 measures. Those tracts that have a statistically significant difference ($p < 0.05$) between the Control and Intervention metrics are signified by ***.

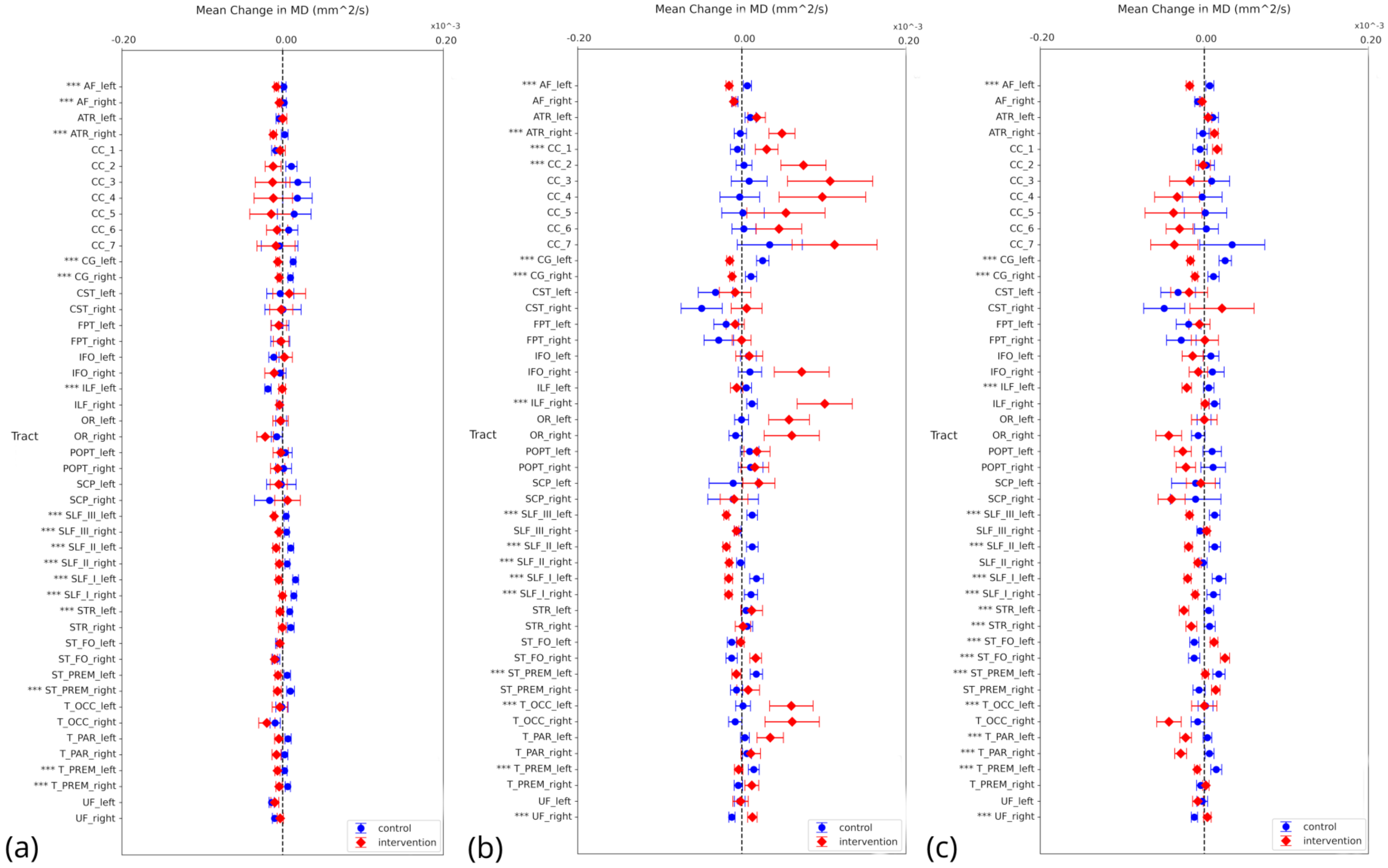

Supplementary Figure 3: Variation in MD between day 1 (a), day 90 (b) and day (180), relative to day 0 measures. Those tracts that have a statistically significant difference ($p < 0.05$) between the Control and Intervention metrics are signified by ***.

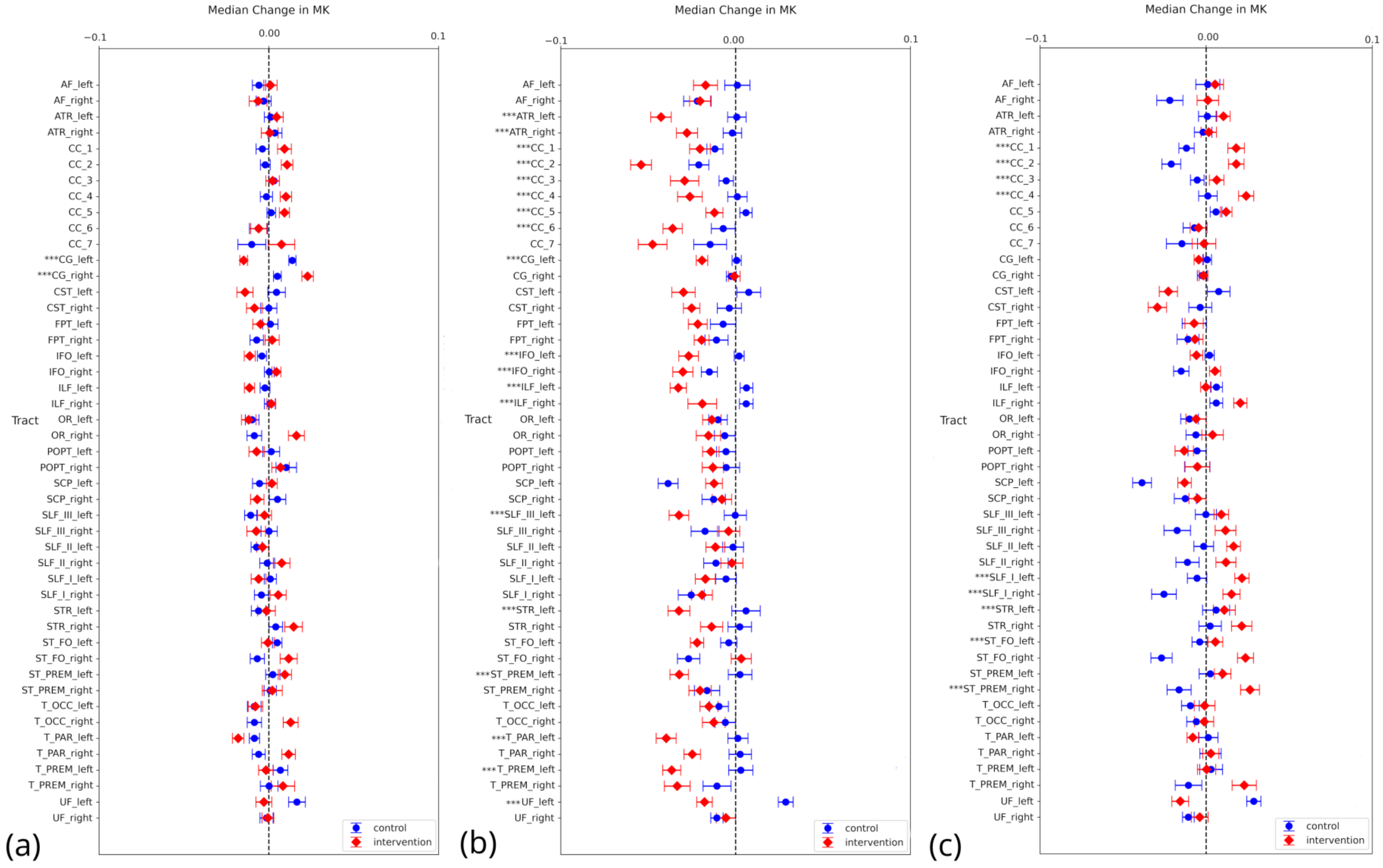

Supplementary Figure 4: Variation in MK between day 1 (a), day 90 (b) and day (180), relative to day 0 measures. Those tracts that have a statistically significant difference ($p < 0.05$) between the Control and Intervention metrics are signified by ***.

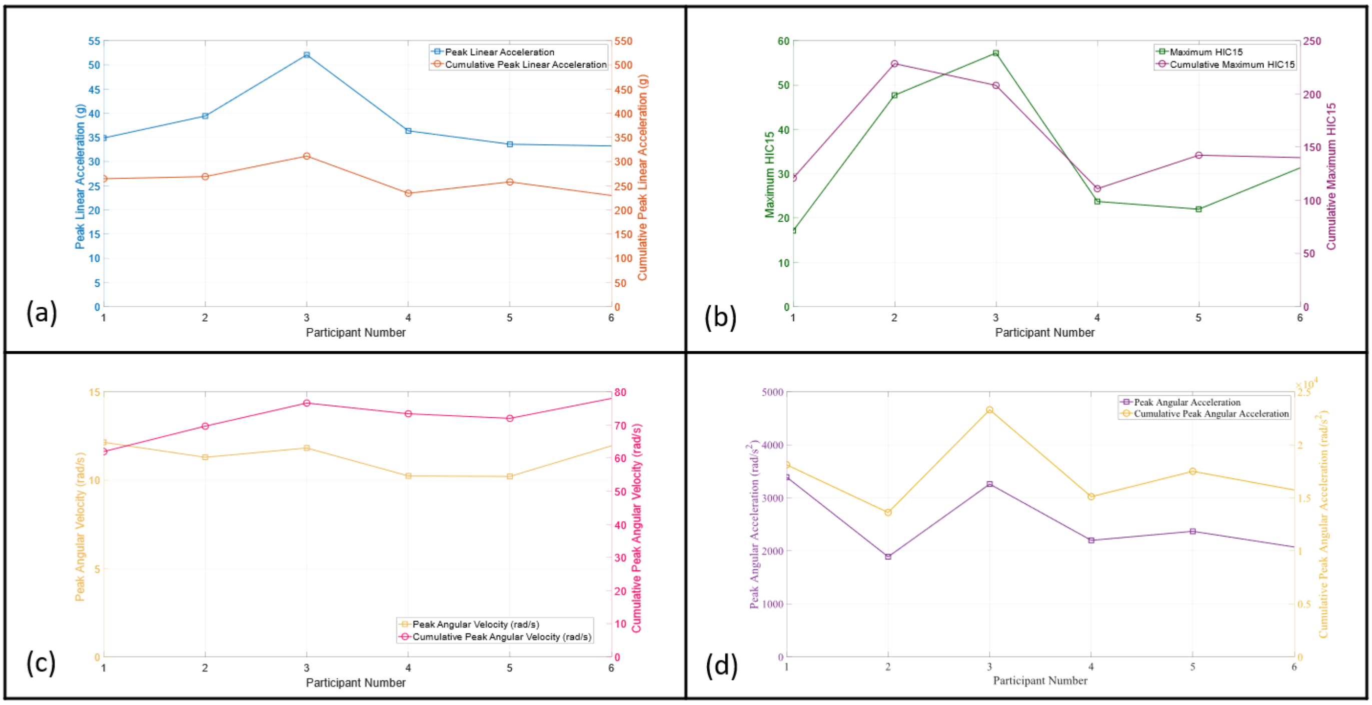

Supplementary Figure 5

The peak kinematics reported for each Intervention participant. (a) Peak linear acceleration (blue) and cumulative PLA (orange); (b) Peak and cumulative head injury criterion scores, calculated over a 15 ms window about the peak acceleration; (c) Peak angular velocity and cumulative PAV; (d) Peak angular acceleration and cumulative PAA.

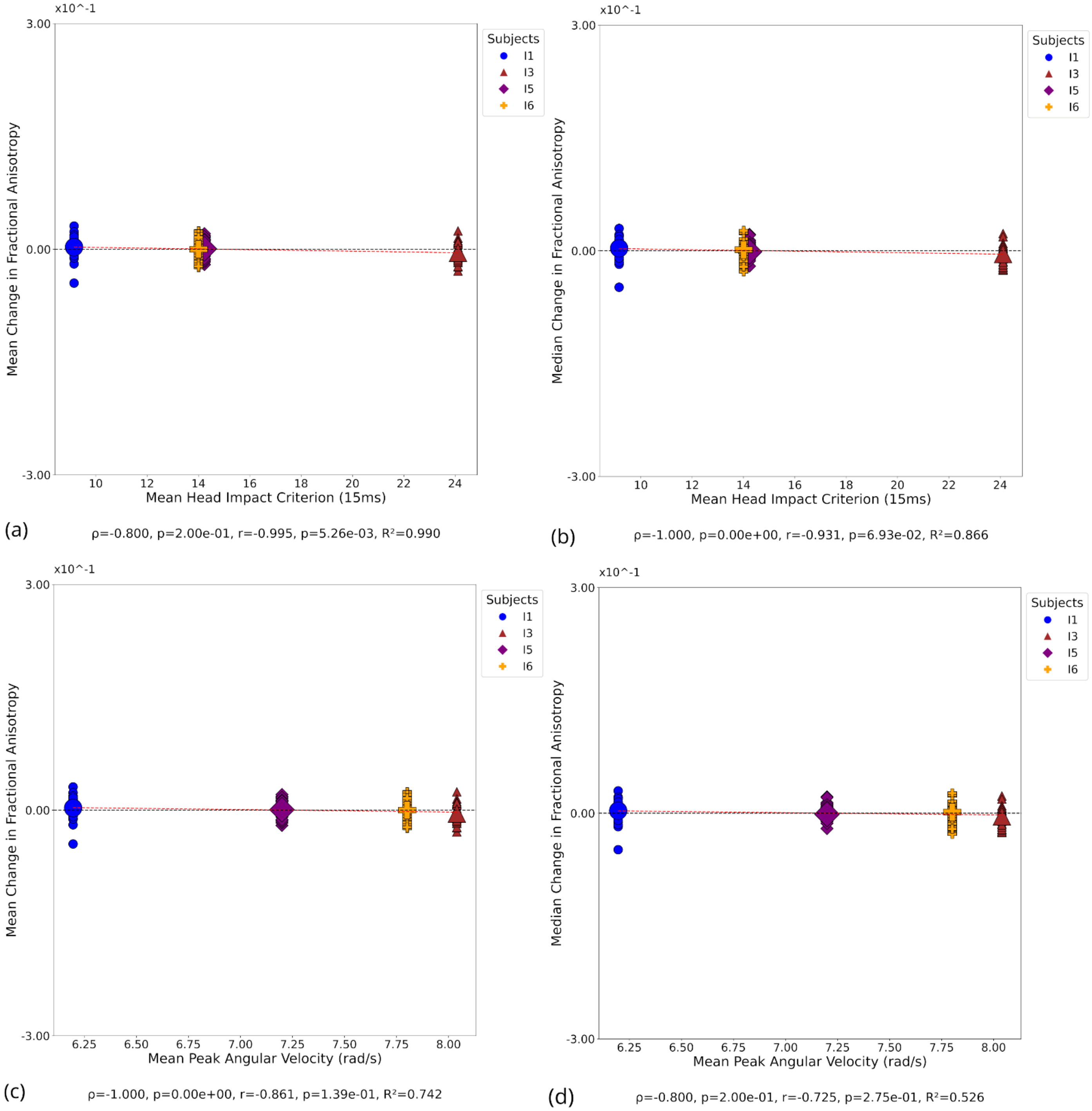

Supplementary Figure 6

Correlation with FA at day 180. Small symbols indicate change in FA in individual white matter tracts at the participant level; large symbols indicate overall change in FA for each participant**.** Pearson's correlation (r), Spearman's correlation (ρ), and coefficient of determination (R^2). **(a)** Mean change in FA versus mean HIC. **(b)** Median change in FA versus mean HIC. **(c)** Mean change in FA versus mean PAV. **(d)** Median change in FA versus mean PAV.

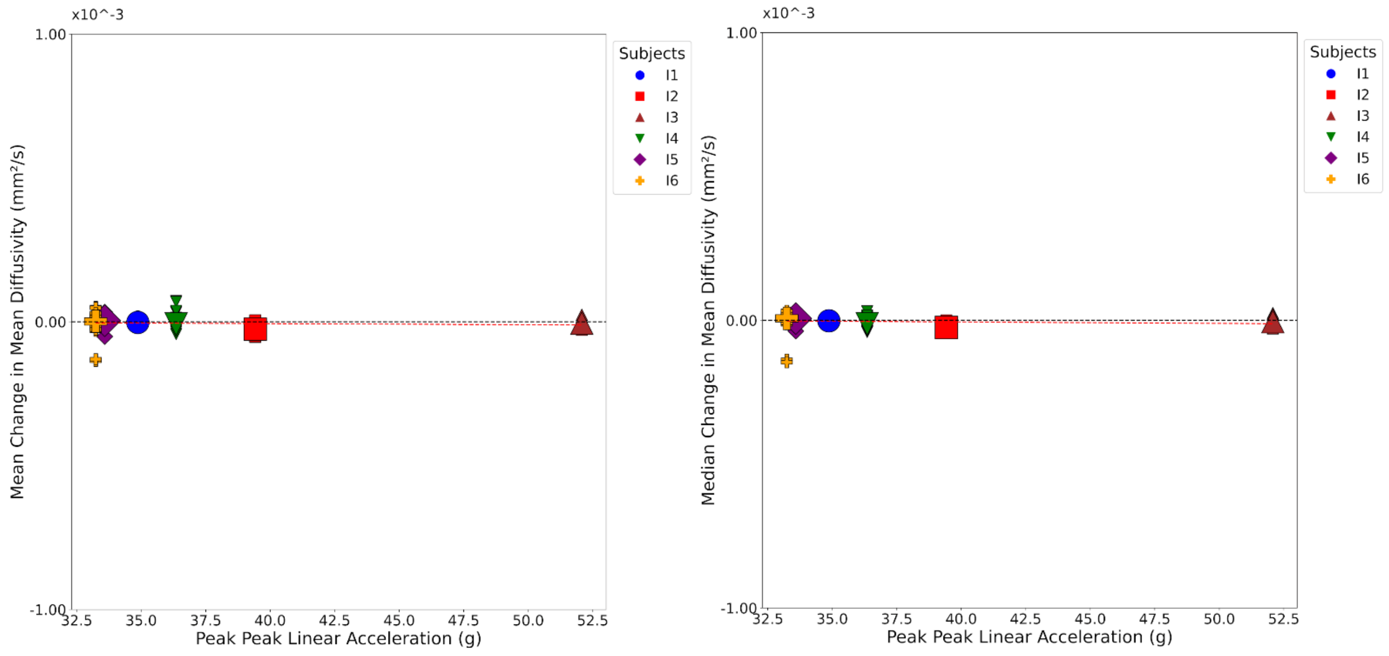

(a) ρ=-0.771, p=7.24e-02, r=-0.256, p=6.24e-01, R^2=0.066

(b) ρ=-0.829, p=4.16e-02, r=-0.299, p=5.65e-01, R^2=0.089

Supplementary Figure 7

Correlation with MD at day 1. Small symbols indicate change in MD in individual white matter tracts at the participant level; large symbols indicate overall change in MD for each participant**.** Pearson's correlation (r), Spearman's correlation (ρ), and coefficient of determination (R^2). **(a)** Mean change in MD versus mean HIC. **(b)** Median change in MD versus mean HIC. **(c)** Mean change in MD versus mean PAV. **(d)** Median change in MD versus mean PAV.

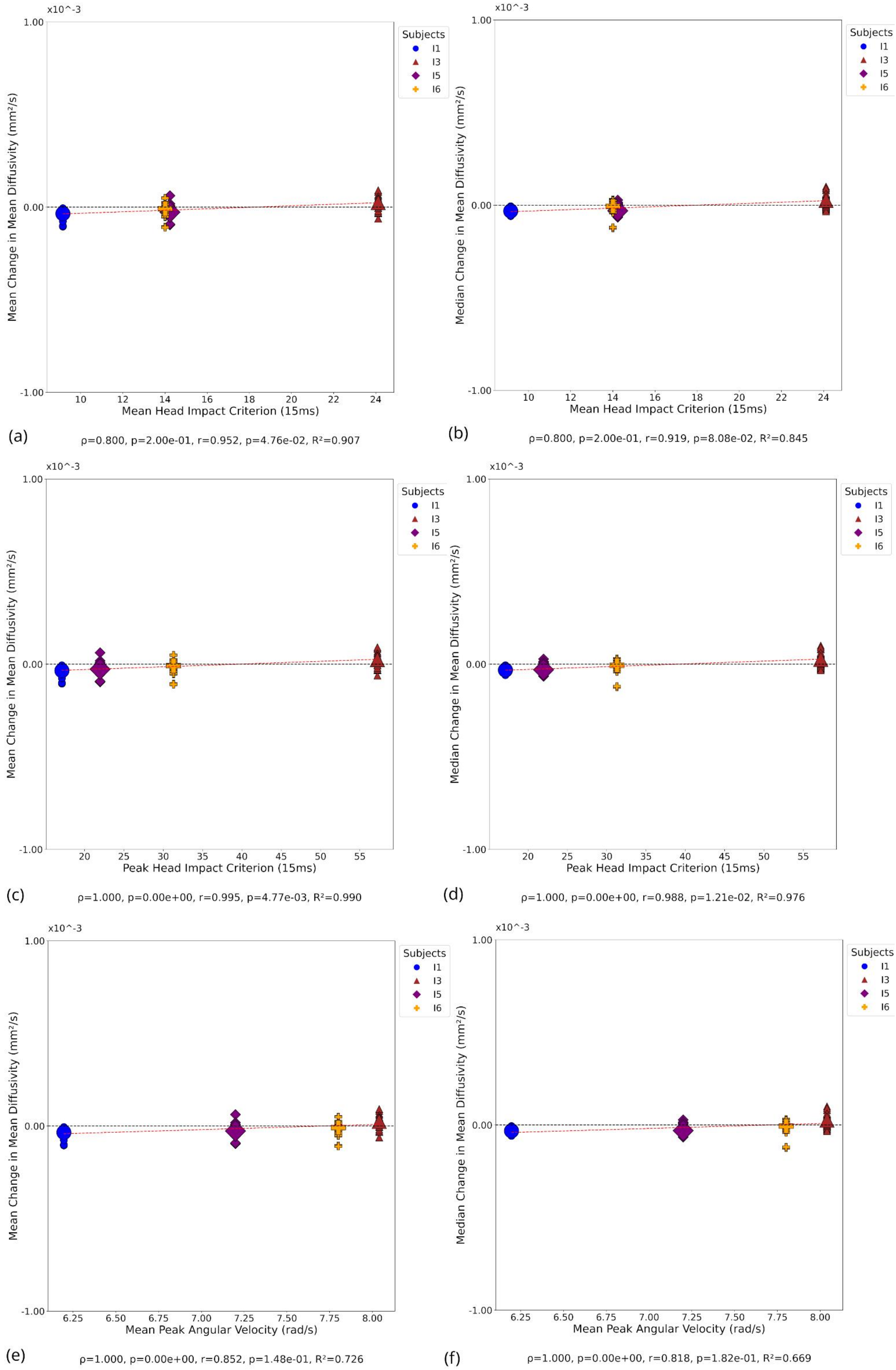

x10^-3
1.00
0.00
-1.00
Mean Change in Mean Diffusivity (mm²/s)
10 12 14 16 18 20 22 24
Mean Head Impact Criterion (15ms)
Subjects
I1
I3
I5
I6
(a)
ρ=0.800, p=2.00e-01, r=0.952, p=4.76e-02, R²=0.907
Median Change in Mean Diffusivity (mm²/s)
Mean Head Impact Criterion (15ms)
(b)
ρ=0.800, p=2.00e-01, r=0.919, p=8.08e-02, R²=0.845
20 25 30 35 40 45 50 55
Peak Head Impact Criterion (15ms)
(c)
ρ=1.000, p=0.00e+00, r=0.995, p=4.77e-03, R²=0.990
(d)
ρ=1.000, p=0.00e+00, r=0.988, p=1.21e-02, R²=0.976
6.25 6.50 6.75 7.00 7.25 7.50 7.75 8.00
Mean Peak Angular Velocity (rad/s)
(e)
ρ=1.000, p=0.00e+00, r=0.852, p=1.48e-01, R²=0.726
(f)
ρ=1.000, p=0.00e+00, r=0.818, p=1.82e-01, R²=0.669

Supplementary Figure 8:

Correlation with MD at day 180. Small symbols indicate change in MD in individual white matter tracts at the participant level; large symbols indicate overall change in MD for each participant**.** Pearson's correlation (r), Spearman's correlation (ρ), and coefficient of determination (R^2). **(a)** Mean change in MD versus mean HIC. **(b)** Median change in MD versus mean HIC. **(c)** Mean change in MD versus peak HIC. **(d)** Median change in MD versus peak HIC. **(e)** Mean change in MD versus mean PAV. **(f)** Median change in MD versus mean PAV.

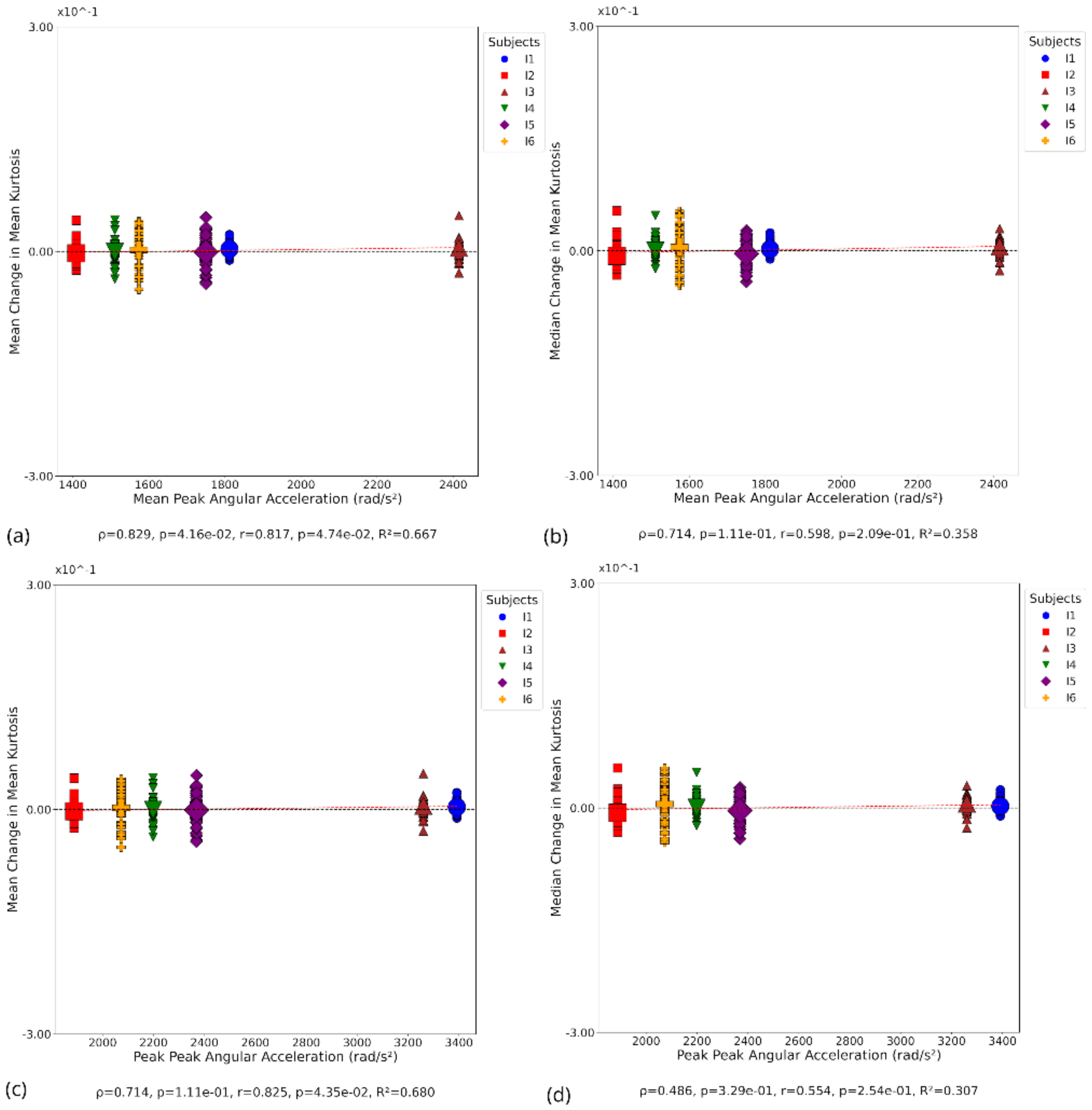

Supplementary Figure 9

Correlation with MK at day 180. Small symbols indicate change in MD in individual white matter tracts at the participant level; large symbols indicate overall change in MK for each participant**.** Pearson's correlation (r), Spearman's correlation (ρ), and coefficient of determination (R^2). **(a)** Mean change in MK versus mean PAA. **(b)** Median change in MK versus mean PAA. **(c)** Mean change in MK versus peak PAA. **(d)** Median change in MK versus peak PAA.
