# Supplementary material for: Quantified Head-Ball Impacts in Soccer: A Preliminary, Prospective Study
Source: Neurotrauma Rep. 2025 Sep 25;6(1):928–43. doi: 10.1177/2689288X251380145 (PMC12547406; doi:10.1177/2689288X251380145)
Supplement: Supplementary Table S1 [file 2689288x251380145_supplementary_table_s1.pdf]

Supplementary Table

Supplementary Table 1: A list of the TractSeg labels and associated anatomical tract names.

AF_left	Arcuate fascicle left
AF_right	Arcuate fascicle right
ATR_left	Anterior Thalamic Radiation
ATR_right	Anterior Thalamic Radiation right
CC_1	Corpus Callosum Rostrum
CC2	Corpus Callosum Genu
CC3 (Premotor)	Corpus Callosum Rostral body (Premotor)
CC4	Corpus Callosum Anterior midbody (Primary Motor)
CC5	Corpus Callosum Posterior midbody (Primary Somatosensory)
CC6	Corpus Callosum Isthmus
CC7	Corpus Callosum Splenium
CG_left	Cingulum left
CG_right	Cingulum right
CST_left	Corticospinal tract left
CST_right	Corticospinal tract right
FPT_left	Fronto-pontine tract left
FPT_right	Fronto-pontine tract right
ICP_left	Inferior cerebellar peduncle left
ICP_right	Inferior cerebellar peduncle right
IFO_left	Inferior occipito-frontal fascicle left
IFO_right	Inferior occipito-frontal fascicle right
ILF_left	Inferior longitudinal fascicle left
ILF_right	Inferior longitudinal fascicle right
MCP	Middle cerebellar peduncle
OR_left	Optic radiation left
OR_right	Optic radiation right
POPT_left	Parieto-occipital pontine left
POPT_right	Parieto-occipital pontine right
SCP_left	Superior cerebellar peduncle left
SCP_right	Superior cerebellar peduncle right
SLF_I_left	Superior longitudinal fascicle I left
SLF_I_right	Superior longitudinal fascicle I right
SLF_II_left	Superior longitudinal fascicle II left
SLF_II_right	Superior longitudinal fascicle II right
SLF_III_left	Superior longitudinal fascicle III left
SLF_III_right	Superior longitudinal fascicle III right
STR_left	Superior Thalamic Radiation left
STR_right	Superior Thalamic Radiation right
UF_left	Uncinate fascicle left
UF_right	Uncinate fascicle right
T_PREM_left	Thalamo-premotor left
T_PREM_right	Thalamo-premotor right
T_PAR_left	Thalamo-parietal left
T_PAR_right	Thalamo-parietal right
T_OCC_left	Thalamo-occipital left
T_OCC_right	Thalamo-occipital right
ST_FO_left	Striato-fronto-orbital left
ST_FO_right	Striato-fronto-orbital right
ST_PREM_left	Striato-premotor left
ST_PREM_right	Striato-premotor right
ST_PAR_left	Striato-parietal left
ST_PAR_right	Striato-parietal right
ST_OCC_left	Striato-occipital left
ST_OCC_right	Striato-occipital right
